# ﻿The first record of *Tricholathys* Chamberlin & Ivie, 1935 (Araneae, Dictynidae) from China, with a new combination and descriptions of seven new species

**DOI:** 10.3897/zookeys.1185.107005

**Published:** 2023-11-29

**Authors:** Lu-Yu Wang, Xian-Jin Peng, Zhi-Sheng Zhang

**Affiliations:** 1 Key Laboratory of Eco-environments in Three Gorges Reservoir Region (Ministry of Education), School of Life Sciences, Southwest University, Chongqing 400715, China Southwest University Chongqing China; 2 College of Life Sciences, Hunan Normal University, Changsha 410081, Hunan, China Hunan Normal University Changsha China

**Keywords:** Description, DNA barcodes, mesh web spiders, morphology, taxonomy, Tricholathysinae

## Abstract

The genus *Tricholathys*, found for the first time in China, is surveyed and seven new species, *T.burangensis***sp. nov.** (♂♀, Thibet), *T.chenzhenningi***sp. nov.** (♂♀, Qinghai), *T.hebeiensis***sp. nov.** (♀, Hebei), *T.lhunzeensis***sp. nov.** (♂♀, Tibet), *Tricholathysrelictoides***sp. nov.** (♂♀, Xinjiang), *T.serrata***sp. nov.** (♂♀, Tibet), and *T.xizangensis***sp. nov.** (♂♀, Tibet), are described. A new combination is proposed for *Tricholathysalxa* (Tang, 2011) **comb. nov.**, ex. *Argenna* Thorell, 1870. Descriptions of all new species are provided, together with digital images, illustrations, and a distribution map. The DNA barcode information of four recently collected species is also provided.

## ﻿Introduction

The mesh-web spider genus *Tricholathys* Chamberlin & Ivie, 1935 is a lesser noticed spider group originally described from North America and last revised by Chamberlin and Gertsch (1958). Ovtchinnikov (1989, 2001) and Marusik et al. (2017) described two species from Central Asia and the Northern Caucasus. Presently, there are 12 species known from the Holarctic (Nearctic, Northern Caucasus, Kyrgyzstan, and Tajikistan) (WSC 2023), but the genus has never been recorded from China.

Here, we record *Tricholathys* from China for the first time, describe seven new species, and transfer to this genus one species. The goal of this paper is to provide detail description of new species and to provide a new combination for a misplaced species.

## ﻿Materials and methods

**Figure 1.**
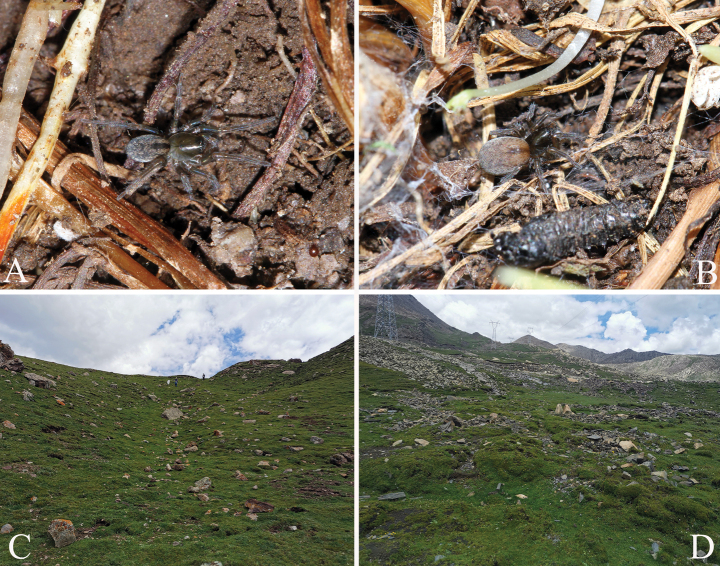
Photos of living specimens and living environment **A–C***Tricholathyschenzhenningi* sp. nov. **A** male **B** female **C** collection site **D***Tricholathyslhunzeensis* sp. nov. collection site.

Photos of all the species presented here were taken with a Canon EOS 7D camera with attached EF 100 mm F2.8L lens (Fig. 1A, B) and Huawei P30 smart phone (Fig. 1C, D). All specimens are preserved in 75% ethanol and were examined, illustrated, photographed, and measured using a Leica M205A stereomicroscope equipped with a drawing tube, a Leica DFC450 camera, and LAS software (v. 4.6). Male palps and epigynes were examined and illustrated after they were dissected. Epigynes were cleared immersing them in pancreatin (Álvarez-Padilla and Hormiga 2007). Eye sizes were measured as the maximum dorsal diameter. Leg measurements are shown as total length (femur, patella and tibia, metatarsus, tarsus). All measurements are in millimetres. Specimens examined here are deposited in the Collection of Spiders, School of Life Sciences, Southwest University, Chongqing, China (**SWUC**).

Terminology follows Marusik et al. (2017). Abbreviations used in the text: **ALE** – anterior lateral eye; **AME** – anterior median eye; **PLE** – posterior lateral eye; **PME** – posterior median eye.

The blood/cell/tissue genomic DNA extraction kit (Tiangen, Beijing) was used to extract genomic DNA from the muscle tissues of legs. The PCR primer for a partial fragment of the mitochondrial cytochrome oxidase subunit I (CO1) gene was the universal primer for invertebrate DNA barcoding LCO1490 (5'-GGTCAACAAATCATAAAGATATTGG-3') (Folmer et al. 1994) and HCOoutout (5'-GTAAATATATGRTGDGCTC-3') (Schulmeister et al. 2002). All sequences were analyzed using BLAST and are deposited in GenBank.

## ﻿Results

### ﻿DNA barcodes

The accession numbers of the generated DNA barcodes are provided in Table 1. The K2P distance of intraspecific and interspecific nucleotide divergences are shown in Table 2.

**Table 1.** Voucher specimen information.

Species	Sex	GenBank accession no.	Sequence length	Collection localities
*T.burangensis* sp. nov.	Male	OK001967	696	Kejia Village, Burang Town, Burang County, Tibet, China
*T.burangensis* sp. nov.	Female	OK001966	696	Kejia Village, Burang Town, Burang County, Tibet, China
*T.chenzhenningi* sp. nov.	Female	OK001968	766	Liuhuanggou, Huangcheng Township, Menyuan County, Qinghai, China
*T.lhunzeensis* sp. nov.	Male	OK001970	699	Ritang Township, Lhunze county, Tibet, China
*T.lhunzeensis* sp. nov.	Female	OK001969	693	Ritang Township, Lhunze county, Tibet, China
*T.xizangensis* sp. nov.	Female	OK001971	708	Meiduo Village, Qulho Township, Coqen County, Tibet, China

**Table 2.** Intraspecific and interspecific nucleotide divergences for four *Tricholathys* species, using Kimura’s two-parameter model.

	Sex	1	2	3	4	5	6
1. *T.xizangensis*	male						
2. *T.lhunzeensis*	male	0.066					
3. *T.lhunzeensis*	female	0.065	0.000				
4. *T.chenzhenningi*	female	0.086	0.072	0.072			
5. *T.burangensis*	male	0.077	0.064	0.063	0.043		
6. *T.burangensis*	female	0.079	0.063	0.063	0.043	0.001	

The intraspecific genetic distance ranged from 0 to 0.1% and the interspecific genetic distance ranged from 4.3% (between *T.chenzhenningi* (female) and *T.burangensis* (male)) to 8.6% (*T.xizangensis* (male) and *T.chenzhenningi* (female)).

### ﻿Taxonomy

**Family Dictynidae O. Pickard-Cambridge, 1871** (卷叶蛛科)

**Subfamily Tricholathysinae Lehtinen, 1967** (毛隐蛛亚科)

#### ﻿Genus Tricholathys

Taxon classificationAnimaliaAraneaeDictynidae

Chamberlin & Ivie, 1935 (毛隐蛛属)

C620195A-6775-5476-A2BD-147A132C8851

##### Type species.

*Tricholathysspiralis* Chamberlin & Ivie, 1935 from Canada and USA.

##### Diagnosis.

*Tricholathys* is most similar to *Arctella* Holm, 1945 in having coiled posterior arm of conductor, but differs from the latter by the wide, twisted, ribbon-like terminal part of the conductor, the tapering, spiraled tip of the conductor, the sclerotized and subcircular coils (except *T.serrata*) of copulatory ducts (Marusik et al. 2017).

##### Description.

Habitus (Figs 1A, B, 3A, B, 5A, B, 7A, 9A, B, 11A, B, 13A, B, 15A, B). Medium-sized spiders (3.0–8.0). Carapace brown to dark brown. Fovea longitudinal. Cervical groove and radial furrows distinct. Chelicerae elongate, brown, with 3–5 promarginal and 2 or 3 retromarginal teeth. Labium and endites brown, longer than wide. Sternum brown and scutellate, with sparse, brown setae. Legs brown. Leg formula 4123 or 1423. Abdomen oval, yellow-brown to dark brown, with lanceolate cardiac mark in anterior half, and with black V-shaped markings in posterior half part. Venter of abdomen yellow-brown, with small and undivided cribellum (female) (Figs 5D, 11C) or somewhat reduced cribellum (male) (Fig. 11D).

**Figure 2.**
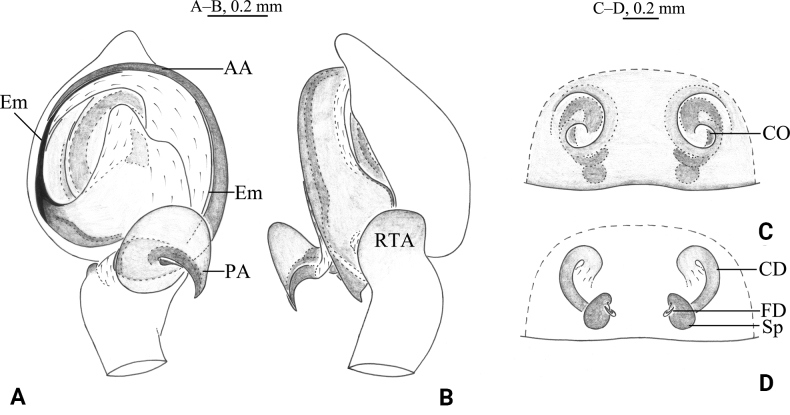
*Tricholathysburangensis* sp. nov. **A, B** holotype male **C, D** paratype female **A** left male palp, ventral view **B** left male palp, retrolateral view **C** epigyne, ventral view **D** epigyne, dorsal view. Abbreviations: AA = anterior arm of conductor; CD = copulatory duct; CO = copulatory opening; Em = embolus; FD = fertilization duct; PA = posterior arm of conductor; RTA = retrolaterial tibial apophysis; Sp = spermatheca.

**Figure 3.**
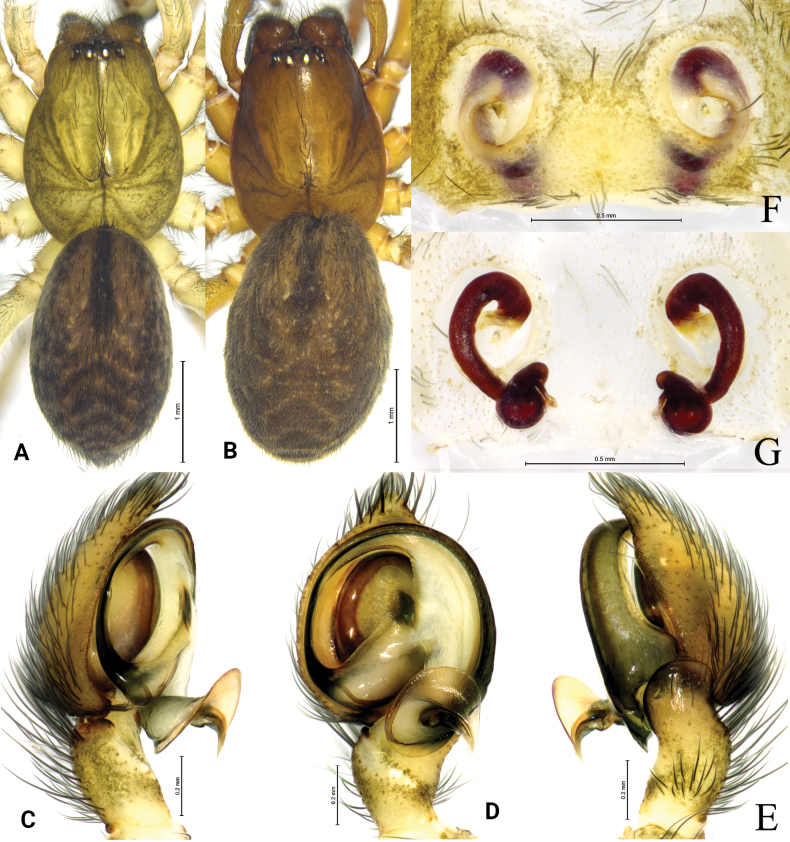
*Tricholathysburangensis* sp. nov. **A, C–E** holotype male **B, F, G** paratype female **A** male habitus, dorsal view **B** female habitus, dorsal view **C** left male palp, prolateral view **D** same, ventral view **E** same, retrolateral view **F** epigyne, ventral view **G** same, dorsal view.

**Figure 4.**
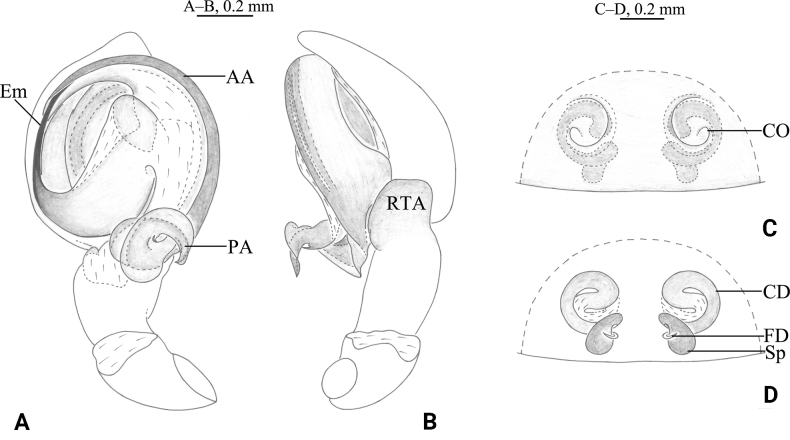
*Tricholathyschenzhenningi* sp. nov. **A, B** holotype male **C, D** paratype female **A** left male palp, ventral view **B** same, retrolateral view **C** epigyne, ventral view **D** same, dorsal view. Abbreviations: AA = anterior arm of conductor; CD = copulatory duct; CO = copulatory opening; Em = embolus; FD = fertilization duct; PA = posterior arm of conductor; RTA = retrolaterial tibial apophysis; Sp = spermatheca.

**Figure 5.**
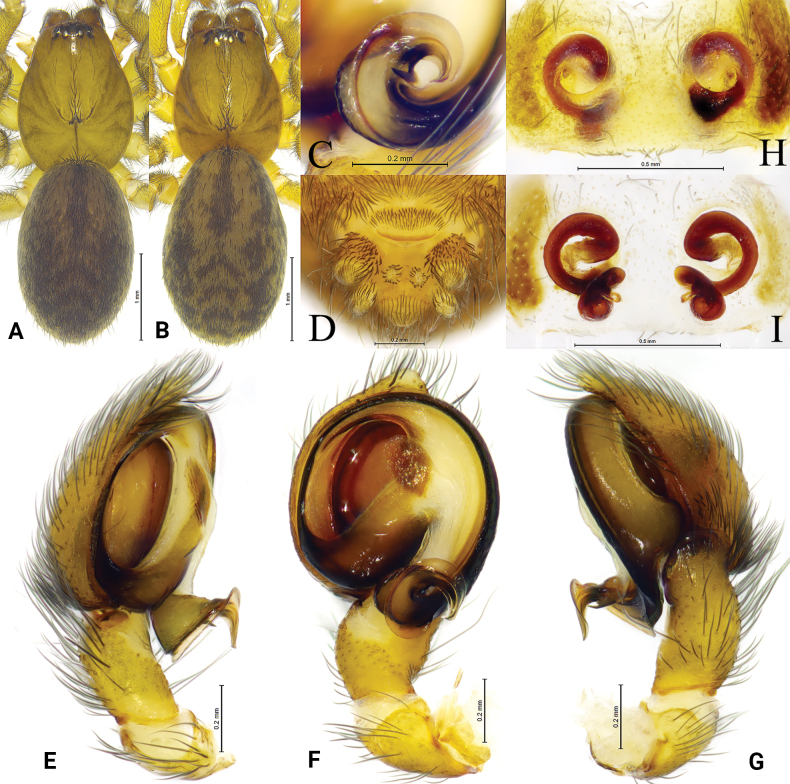
*Tricholathyschenzhenningi* sp. nov. **A, C–G** holotype male **B, H, I** paratype female **A** male habitus, dorsal view **B** female habitus, dorsal view **C** posterior arm of conductor, ventral view **D** cribellum, ventral view **E** left male palp, prolateral view **F** same, ventral view **G** same, retrolateral view **H** epigyne, ventral view **I** same, dorsal view.

Male palp (Figs 2A, B, 3C–E, 4A, B, 5C, E–G, 8A, B, 9C–E, 10A, B, 11E–G, 12A, B, 13C–E, 14A, B, 15C–E) with broad, retrolateral tibial apophysis. Cymbium slightly longer than wide; bulb as long as wide. Conductor with 2 well-developed arms: anterior arm tapering gradually and extend towards base of embolus; posterior arm terminating in spiral and with digitiform process (DP in Figs 14B, 15E; mostly covered by retrolateral tibial apophysis). Embolus long and extended clockwise, distal part thread-like and hidden by conductor.

Epigyne (Figs 2C, D, 3F, G, 4C, D, 5H, I, 6A, B, 7B, C, 8C, D, 9F, G, 10C, D, 11H, I, 12C, D, 13F, G, 14C, D, 15F, G). Copulatory openings cochleate. Copulatory ducts with 2 distinct parts: weakly sclerotized part connected to copulatory openings and a strongly sclerotized part forming almost an entire circle (except *T.serrata* sp. nov.). Spermathecae nearly globular and small, less than the distance between spermathecae. Fertilization ducts small, hook-shaped.

##### Composition.

Eighteen species (WSC 2023 and the data first presented herein).

##### Distribution.

The genus has a distinctive range and is known from the Nearctic, eastern part of Central Asia (Kyrgyzstan and Tajikistan), western and northern part of China, and the Northern Caucasus.

##### Habitats.

Judging from our collection, species of *Tricholathys* prefer to live in high-elevation habitats near rivers or at the snow line, building small mesh-webs under stones.

#### ﻿ Tricholathys alxa

Taxon classificationAnimaliaAraneaeDictynidae

(Tang, 2011), comb. nov. (阿拉善毛隐蛛)

4146309F-A89F-501D-BB3D-6830FCD6056A

Argenna alxa Tang, 2011: 94, fig. A–D. (Male holotype and two female paratypes from Mt. Helanshan, Alxa Left County, Alxa League (City), Inner Mongolia of China, deposited in the College of Life Sciences and Technology, Inner Mongolia Normal University, China.).

##### Notes.

The types of *Argennaalxa* Tang, 2011 were unavailable for our study, but it is clear from the description and figures by Tang (2011: 94, figs A–D) that it has broad retrolateral tibial apophysis, the posterior arm of conductor terminating in a spiral, rounded mesal copulatory opening margins, relatively long copulatory ducts, and small, globular spermathecae. So, it is definitely a species of *Tricholathys*, not a species of *Argenna*. Here, we formally transfer it into *Tricholathys*.

Judging from the original illustrations, the species is much similar to *T.lhunzeensis* sp. nov. (Figs 8A–D, 9C–G), but *T.alxa* comb. nov. can be distinguished by the wider posterior arm of conductor and the end of conductor slightly curved retrolaterally, other than curved retro-proximally and the narrower space between copulatory openings and the different shape of copulatory ducts and spermathecae.

##### Distribution.

China (Alxa of Inner Mongolia).

#### ﻿ Tricholathys burangensis

Taxon classificationAnimaliaAraneaeDictynidae

sp. nov. (普兰毛隐蛛)

9B0DB687-465A-58C9-95C6-E322429B7D7E

https://zoobank.org/F3816ED3-9A9D-4D0D-9A5C-697D1E0184F6

Figs 2 , 3 , 16

##### Type materials.

***Holotype* male**: China, Tibet, Burang County, Burang Town, Kejia Village, 30°11′17.48′′N, 81°16′21.66′′E, elev. 3685 m; 24 July 2020, L.Y. Wang et al. leg. (SWUC-T-DI-07-01). ***Paratypes***: 2 males and 13 females (SWUC-T-DI-07-02~16), with same data as holotype.

##### Etymology.

The specific name is derived from the county where the type locality is located; it is used as a noun in apposition.

##### Diagnosis.

The male of this new species is similar to *T.subnivalis* (Ovtchinnikov, 1989) (Marusik et al. 2017: 256, fig. 4D–F) in having the embolus originating at about 7:30 o’clock, the anterior arm of the conductor gradually tapering and terminating at about 9 o’clock, the posterior arm of the conductor wide and spiral; the new species differs from *T.subnivalis* in having the posterior arm of the conductor with a pointed end (hook-shaped in *T.subnivalis*) and retrolateral tibial apophysis crooked (straight in *T.subnivalis*) (Figs 2A, B, 3C–E). The female of the new species is similar to that of *T.ovtchinnikovi* Marusik, Omelko & Ponomarev, 2017 (Marusik et al. 2017: 258, fig. 6J–L) in having the copulatory ducts semicircular, spermathecae globular, and fertilization ducts thin and hook-shaped, but the new species differs from *T.ovtchinnikovi* in having the copulatory ducts widely spaced and the strongly sclerotized part is five times longer than the length of weakly sclerotized part (vs 2.5 times longer in *T.ovtchinnikovi*).

##### Description.

**Male (holotype).** Habitus as in Fig. 3A. Total length 4.24 (4.39–4.46 in male paratypes). Prosoma 2.08 long, 1.61 wide; opisthosoma 2.36 long, 1.42 wide. Eye sizes and interdistances: AME 0.07, ALE 0.09, PME 0.07, PLE 0.09; AME–AME 0.06, AME–ALE 0.06, PME–PME 0.11, PME–PLE 0.11, ALE–PLE 0.05. MOA 0.23 long, anterior width 0.21, posterior width 0.26. Clypeus height 0.11. Chelicerae with 4 promarginal and 3 retromarginal teeth. Leg measurements: I 4.89 (1.48, 1.71, 0.98, 0.72); II 4.18 (1.22, 1.45, 0.85, 0.66); III 3.50 (1.03, 1.17, 0.73, 0.57); IV 5.20 (1.51, 1.78, 1.19, 0.72). Leg formula: 4123.

***Palp*** (Figs 2A, B, 3C–E). Tibia with broad and truncate retrolateral apophysis, S-shaped, its width slightly less than the length of tibia. Tip of cymbium with 4 spines. Anterior arm of conductor (AA) tapering gradually and terminating at about 9:00 o’clock position; posterior arm (PA) terminating in spiral with sharply pointed tip, subterminal part with distinct extension. Embolus originating at about 7:30 o’clock position.

**Female paratype.** Habitus as in Fig. 3B. Total length 4.54 (4.54–5.05 in other female paratypes). Carapace 2.19 long, 1.68 wide; opisthosoma 2.72 long, 1.76 wide. Eye sizes and interdistances: AME 0.08, ALE 0.10, PME 0.08, PLE, 0.11; AME-AME 0.08, AME-ALE 0.07, PME-PME 0.12, PME-PLE 0.13, ALE-PLE 0.04. MOA 0.25 long, anterior width 0.26, posterior width 0.29. Clypeus 0.15 high. Leg measurements: I 4.64 (1.33, 1.63, 1.02, 0.66); II 3.90 (1.11, 1.29, 0.87, 0.63); III 3.68 (1.11, 1.16, 0.84, 0.57); IV 4.95 (1.45, 1.66, 1.14, 0.70). Leg formula: 4123.

***Epigyne*** (Figs 2C, D, 3F, G). Copulatory openings spiraled and somewhat 6-shaped (right one), spaced by about 3 times of its width. Weakly sclerotized part of copulatory ducts connected to the copulatory openings (trumpet-shaped) and strongly sclerotized part forming semicircular. Space between copulatory ducts wider than space between spermathecae. Spermathecae small, almost comma-shaped, length/width 3/2 and spaced by twice its diameter. Fertilization ducts thin, hook-shaped.

##### Distribution.

Known only from the type locality, Tibet, China (Fig. 16).

#### ﻿ Tricholathys chenzhenningi

Taxon classificationAnimaliaAraneaeDictynidae

sp. nov. (陈氏毛隐蛛)

1A6CA21C-C5FC-5184-9B49-8310F58751E6

https://zoobank.org/268C6596-71B6-4084-B91E-0389369E1985

Figs 1 , 4 , 5 , 16

##### Type materials.

***Holotype* male**: China, Qinghai, Menyuan County, Huangcheng Township, Liuhuanggou, 37°47′50.69″N, 101°16′48.23″E, elev. 3491 m, 13 August 2019, L.Y. Wang and Z.J. Shi leg. (SWUC-T-DI-08-01). ***Paratypes***: 5 males and 5 females (SWUC-T-DI-08-02~11), with same data as holotype; 1 male (SWUC-T-DI-08-12), Qilian County, Ebao Town, Jiangyangling, 37°50′21.27″N, 101°6′47.75″E, elev. 3733 m, L.Y. Wang and Z.J. Shi leg.

##### Etymology.

The specific name is a patronym in honor of Prof. Zhenning Chen from Qinghai Normal University in Xining.

##### Diagnosis.

The new species is similar to *T.ovtchinnikovi* (Marusik et al. 2017: 258, figs 2E, F, 4G–I, 5A–E, 6J–L) in having the embolus originating at about 7:30 o’clock position, the anterior arm of the conductor tapering gradually and terminating at about 9:30 o’clock, the posterior arm of conductor spiral, distal, and with a small tooth at the center, the copulatory ducts semicircular, the spermathecae spherical, and the fertilization ducts thin and hook-shaped; the new species differs from *T.ovtchinnikovi* in having the posterior arm of the conductor wide (narrow in *T.subnivalis*), the retrolateral tibial apophysis fat and as wide as the tibia (slender in *T.subnivalis* and narrower than the tibia) (Figs 4A, B, 5C, E–G), and the copulatory ducts rounded (oval in *T.subnivalis*) (Figs 4C, D, 5H–I).

##### Description.

**Male (holotype).** Habitus as in Fig. 5A. Total length 3.73 (3.40–4.57 in male paratypes). Prosoma 1.77 long, 1.36 wide; opisthosoma 2.12 long, 1.36 wide. Eye sizes and interdistances: AME 0.07, ALE 0.09, PME 0.06, PLE 0.08; AME–AME 0.05, AME–ALE 0.04, PME–PME 0.12, PME–PLE 0.09, ALE–PLE 0.05. MOA 0.20 long, anterior width 0.18, posterior width 0.24. Clypeus height 0.12. Chelicerae with 4 promarginal and 3 retromarginal teeth. Leg measurements: I 4.08 (1.12, 1.55, 0.84, 0.57); II 3.29 (0.95, 1.09, 0.73, 0.52); III 2.92 (0.94, 0.91, 0.63, 0.44); IV 4.26 (1.24, 1.47, 1.00, 0.55). Leg formula: 4123.

***Palp*** (Figs 4A, B, 5C, E–G). Tip of cymbium with shorter tip and 4 spines. Anterior arm of conductor tapering gradually and terminating at about 9:30 o’clock position; posterior arm with small spine (Fig. 5C, F, G; 4A, B). Embolus originating at about 7:30 o’clock position; distal part covered by conductor.

**Female (paratype).** Habitus as in Fig. 5B. Total length 3.84 (3.33–4.72 in other female paratypes). Prosoma 1.74 long, 1.35 wide; opisthosoma 2.37 long, 1.50 wide. Eye sizes and interdistances: AME 0.07, ALE 0.10, PME 0.07, PLE, 0.07; AME–AME 0.07, AME–ALE 0.05, PME–PME 0.11, PME–PLE 0.10, ALE–PLE 0.05. MOA 0.22 long, anterior width 0.20, posterior width 0.25. Clypeus height 0.10. Leg measurements: I 3.78 (1.11, 1.37, 0.75, 0.55); II 3.31 (1.00, 1.12, 0.70, 0.49); III 2.71 (0.81, 0.79, 0.67, 0.44); IV 4.03 (1.17, 1.37, 0.99, 0.50). Leg formula: 4123.

***Epigyne*** (Figs 4C, D, 5H, I). Copulatory openings cochleate, space between them wider than the diameter of the copulatory openings. Weakly sclerotized parts of copulatory ducts S-shaped, and strongly sclerotized part forming almost an entire circle. Spermathecae small, comma-shaped, nearly two times longer than its width. Space between spermathecae slightly narrower than space between copulatory ducts, nearly two times of the width of spermathecae.

##### Distribution.

China (Qinghai).

#### ﻿ Tricholathys hebeiensis

Taxon classificationAnimaliaAraneaeDictynidae

sp. nov. (河北毛隐蛛)

0DC13BA9-59F9-503A-BD80-F63D080D221A

https://zoobank.org/DFFFF45A-3530-4997-AF7E-E182918C8D6E

Figs 6 , 7 , 16

##### Type materials.

***Holotype* female**: China, Hebei Province, Yu County, Xiaowutai Mountains, 39°56′31″N, 114°56′36″E, no detailed GPS data on the locality, 5 June 1998, W.L. Lue leg. (SWUC-T-DI-09-01).

##### Etymology.

The specific name is derived from Hebei Province, where the type locality is located.

##### Diagnosis.

**Figure 6.**
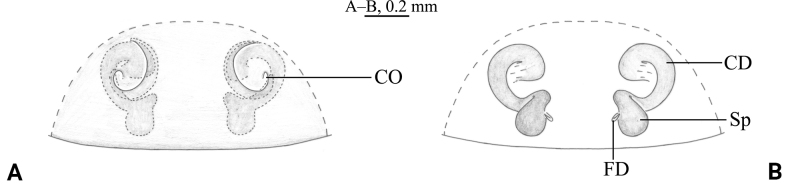
*Tricholathyshebeiensis* sp. nov. holotype female **A** epigyne, ventral view **B** same, dorsal view. Abbreviations: CD = copulatory duct; CO = copulatory opening; FD = fertilization duct; Sp = spermatheca.

**Figure 7.**
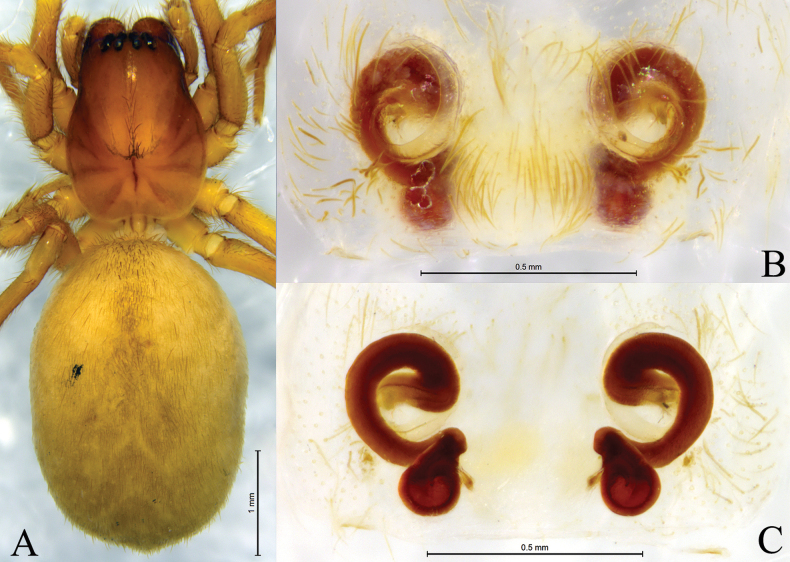
*Tricholathyshebeiensis* sp. nov., holotype female **A** female habitus, dorsal view **B** epigyne, ventral view **C** same, dorsal view.

This new species is similar to *T.burangensis* sp. nov. (Figs 2C, D, 3F, G) in having the copulatory openings cochleate, the copulatory ducts semicircular, the spermathecae comma-shaped, and the fertilization ducts thin and hook-shaped, but it differs from that species in shape of the copulatory ducts, which are semicircular strongly bending and sclerotized (Figs 6A, B, 7B, C).

##### Description.

**Female (holotype).** Habitus as in Fig. 7A. Total length 4.97. Prosoma 1.81 long, 1.32 wide; opisthosoma 3.16 long, 2.14 wide. Eye sizes and interdistances: AME 0.07, ALE 0.10, PME 0.08, PLE 0.08; AME–AME 0.07, AME–ALE 0.05, PME–PME 0.12, PME–PLE 0.09, ALE–PLE 0.05. MOA 0.21 long, anterior width 0.20, posterior width 0.27. Clypeus height 0.10. Chelicerae with 5 promarginal and 3 retromarginal teeth. Leg measurements: I 3.03 (0.93, 1.07, 0.63, 0.40); II 2.62 (0.82, 0.90, 0.56, 0.34); III 2.29(0.69, 0.76, 0.52, 0.32); IV 3.26 (0.97, 1.09, 0.78, 0.42). Leg formula: 4123.

***Epigyne*** (Figs 6A, B, 7B, C). Copulatory openings cochleate. Copulatory ducts e-shaped; strongly sclerotized part forming almost semicircle and 6 times longer than length of weakly sclerotized part. Spermathecae small, nearly pear-shaped; space between spermathecae twice as wide as diameter of spermathecae.

**Male.** Unknown.

##### Distribution.

Known only from the type locality, Hebei, China (Fig. 16).

#### ﻿ Tricholathys lhunzeensis

Taxon classificationAnimaliaAraneaeDictynidae

sp. nov. (隆子毛隐蛛)

1607883E-7667-5F38-8A4C-9ACFDE2B17BA

https://zoobank.org/DB6F7DA0-0851-4CA6-B2CE-DB44E3BC24B2

Figs 8 , 9 , 16

##### Type materials.

***Holotype* male**: China, Tibet, Lhunze county, Ritang Township, 28°37′16.02′′N, 92°13′4.59′′E, elev. 4988 m, 5 August 2020, L.Y. Wang et al. leg. (SWUC-T-DI-10-01). ***Paratypes*** (9 males and 28 females): 9 males and 21 females (SWUC-T-DI-10-02~31), with same data as holotype; 7 females (SWUC-T-DI-10-32~38), same locality with holotype, 28°37′16.05′′N, 92°13′4.99′′E, elev. 4996 m, 3 August 2020, L.Y. Wang, et al. leg.

##### Etymology.

The specific name is derived from the name of the county where the type locality is located.

##### Diagnosis.

**Figure 8.**
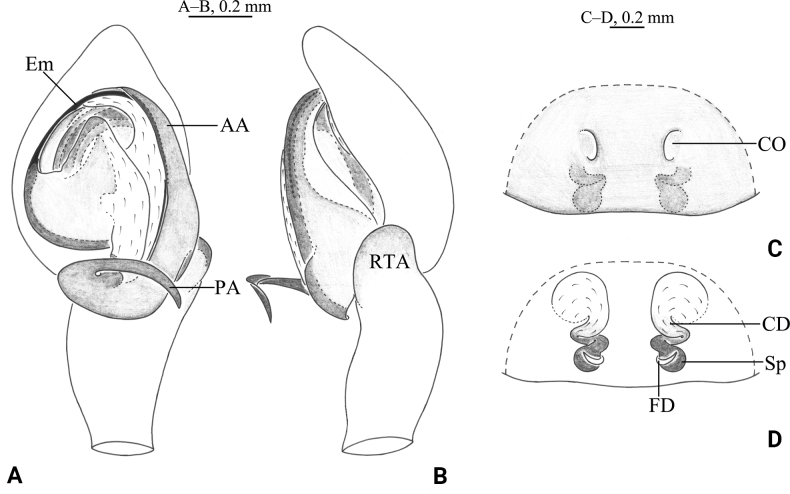
*Tricholathyslhunzeensis* sp. nov. **A, B** holotype male **C, D** paratype female **A** left male palp, ventral view **B** same, retrolateral view **C** epigyne, ventral view **D** same, dorsal view. Abbreviations: AA = anterior arm of conductor; CD = copulatory duct; CO = copulatory opening; Em = embolus; FD = fertilization duct; PA = posterior arm of conductor; RTA = retrolaterial tibial apophysis; Sp = spermatheca.

**Figure 9.**
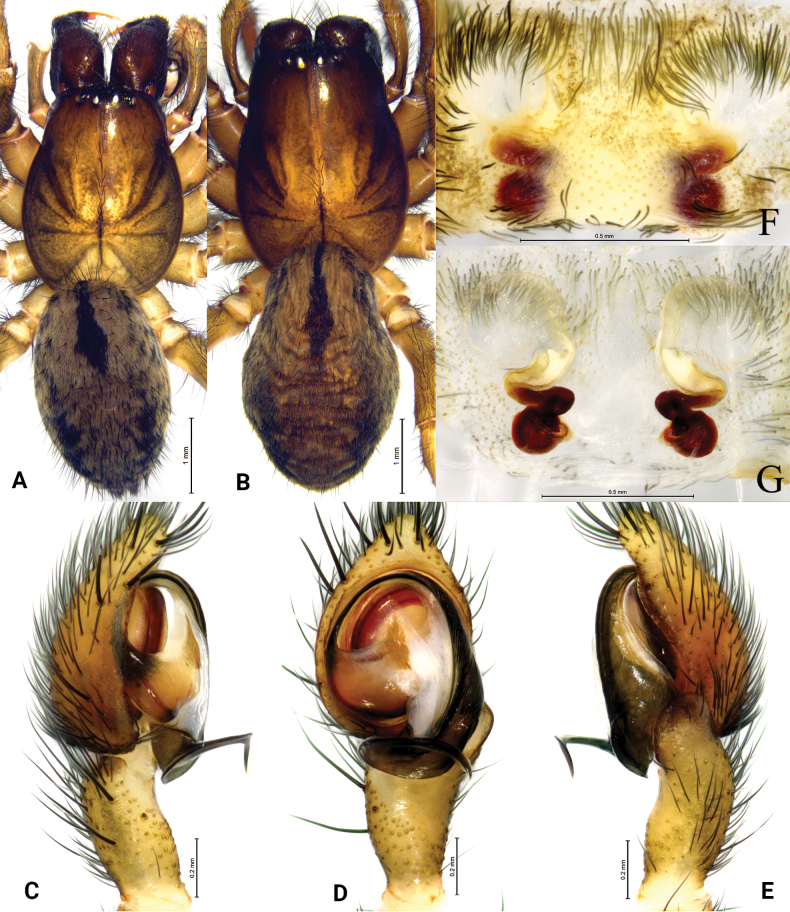
*Tricholathyslhunzeensis* sp. nov. **A, C–E** holotype male **B, F, G** paratype female **A** male habitus, dorsal view **B** female habitus, dorsal view **C** left male palp, prolateral view **D** same, ventral view **E** same, retrolateral view **F** epigyne, ventral view **G** same, dorsal view.

**Figure 10.**
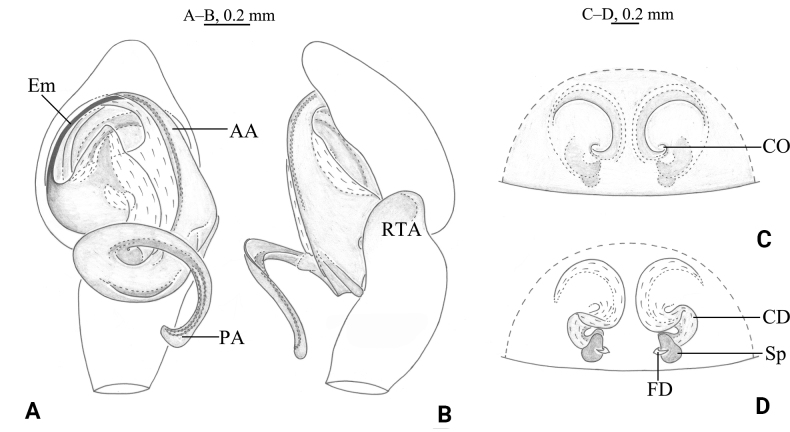
*Tricholathysrelictoides* sp. nov. **A, B** holotype male **C, D** paratype female **A** left male palp, ventral view **B** same, retrolateral view **C** epigyne, ventral view **D** same, dorsal view. Abbreviations: AA = anterior arm of conductor; CD = copulatory duct; CO = copulatory opening; Em = embolus; FD = fertilization duct; PA = posterior arm of conductor; RTA = retrolaterial tibial apophysis; Sp = spermatheca.

**Figure 11.**
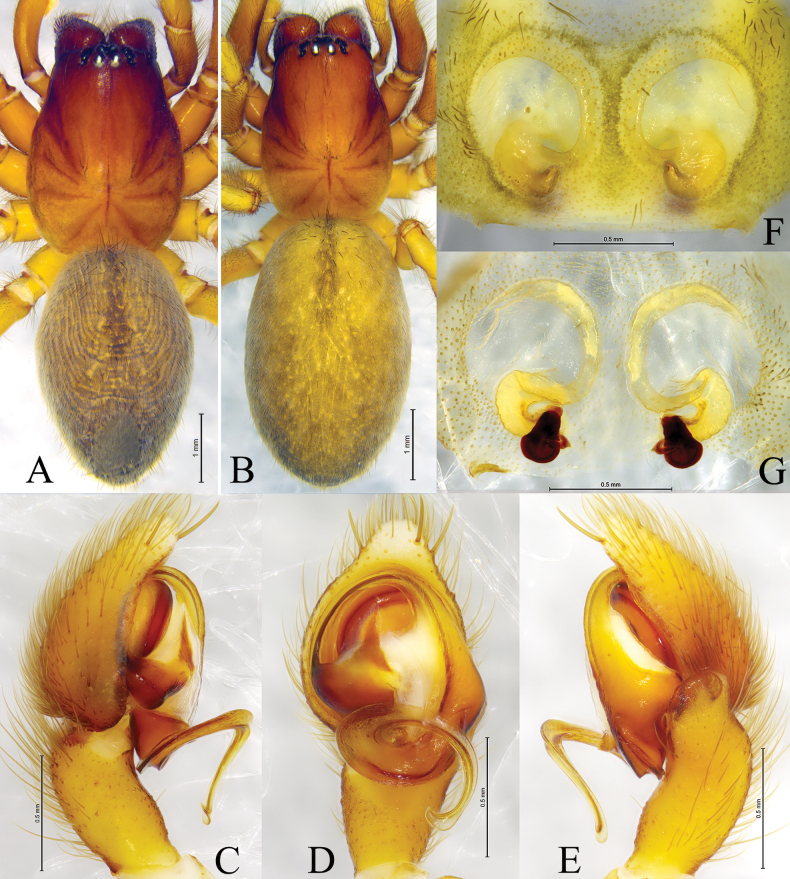
*Tricholathysrelictoides* sp. nov. **A, C–E** holotype male **B, F, G** paratype female **A** male habitus, dorsal view **B** female habitus, dorsal view **C** left male palp, prolateral view **D** same, ventral view **E** same, retrolateral view **F** epigyne, ventral view **G** same, dorsal view.

The new species is similar to *T.relictoides* (Figs 10A–D, 11C–G) in having the embolus originating at about 9:00 o’clock, the anterior arm of conductor gradually tapering and terminating at about the 11:00 o’clock position, the posterior arm of conductor spiral and longer than the anterior arm, and the weakly sclerotized part of the copulatory duct S-shaped; the new species differs from *T.relictoides* in having the posterior arm of conductor short (half of length of embolus) with its end sharp (vs as long as embolus and with hook-shaped tip), the tip of cymbium with 6 spines (vs 3), the base of the embolus differently shaped (Figs 8A, B, 9C–E), and the copulatory openings small, narrow, and with a wide space between them (Figs 8C, D, 9F, G; vs large, wide, and with the space between them narrower than their diameter), weakly sclerotized parts of the copulatory ducts comma-like (vs tube-like).

##### Description.

**Male (holotype).** Habitus as in Fig. 9A. Total length 4.42 (5.35–5.99 in male paratypes). Prosoma 2.91 long, 2.13 wide; opisthosoma 2.85 long, 1.79 wide. Eye sizes and interdistances: AME 0.08, ALE 0.12, PME 0.09, PLE 0.12; AME–AME 0.10, AME–ALE 0.07, PME–PME 0.17, PME–PLE 0.16, ALE–PLE 0.06. MOA 0.31 long, anterior width 0.29, posterior width 0.35. Clypeus height 0.18. Chelicerae with 3 promarginal and 3 retromarginal teeth. Leg measurements: I 6.87 (1.91, 2.52, 1.46, 0.98); II 5.84 (1.73, 2.15, 1.25, 0.71); III 5.24 (1.54, 1.81, 1.10, 0.79); IV 6.98 (1.92, 2.32, 1.74, 1.00). Leg formula: 4123. Cribellum undivided. Dorsum of opisthosoma with black cardiac marking anteriorly.

***Palp*** (Figs 8A, B, 9C–E). Tip of cymbium with six spines. Anterior arm of conductor tapering gradually and terminating at about 11:00 o’clock; posterior arm terminating in spiral, with its distal part narrowed and arc-shaped, almost one-half length of embolus, pointed posteriorly. Embolus originating at about 9:00 o’clock position.

**Female (paratype).** Habitus as in Fig. 9B); total length 6.05 (4.35–6.08 in other paratype females). Prosoma 3.17 long, 2.36 wide; opisthosoma 3.38 long, 2.13 wide. Eye sizes and interdistances: AME 0.10, ALE 0.13, PME 0.11, PLE, 0.13; AME–AME 0.12, AME–ALE 0.09, PME–PME 0.19, PME–PLE 0.17, ALE–PLE 0.06. MOA 0.33 long, anterior width 0.33, posterior width 0.39. Clypeus height 0.21. Leg measurements: I 6.93 (1.99, 2.56, 1.47, 0.91); II 6.15 (1.71, 2.20, 1.33, 0.91); III 5.53 (1.64, 1.81, 1.28, 0.80); IV 7.24 (2.13, 2.49, 1.78, 0.84). Leg formula: 4123.

***Epigyne*** (Figs 8C, D, 9F, G). Copulatory openings oval, with distinct inner margins. Weakly sclerotized part of conductor large, comma-like; strongly sclerotized part S-shaped. Space between copulatory ducts narrower than space between spermathecae. Spermathecae small, nearly globular, widely separated by a space twice the diameter of spermathecae.

##### Distribution.

Known only from the type locality, Tibet, China (Fig. 16).

#### ﻿ Tricholathys relictoides

Taxon classificationAnimaliaAraneaeDictynidae

sp. nov. (类残毛隐蛛)

FB5A0325-AE3B-5422-AB19-6FF5C97E3311

https://zoobank.org/74B37D38-8684-4C7C-9E6C-E38BF50D31BB

Figs 10 , 11 , 16

##### Type materials.

***Holotype* male**: China, Xinjiang, Hami City, Barkol, 43°16′55″N, 93°16′45″E, spruce forests, 9 September 1992, M.J. Song and N.L. Zhou leg. (SWUC-T-DI-11-01). ***Paratypes***: 1 male and 4 females (SWUC-T-DI-10-02~06), with same data as holotype.

##### Etymology.

The specific name is taken from the similarity to *T.relicta* (Ovtchinnikov, 2001).

##### Diagnosis.

This new species is similar to the Central Asian species, *T.relicta* (see Ovtchinnikov 2001: 7, figs 1–4) in having the same number of spines on the tip of cymbium, the embolus originating at about 9:00 o’clock, the anterior arm of the conductor gradually tapering and terminating at about the 11:00 o’clock position, the posterior arm of the conductor spiral and much longer than the anterior arm, the copulatory openings large, with a relatively narrow space between them, and the weakly sclerotized part of the copulatory duct S-shaped; the new species differs from *T.relicta* in having the end of the retrolateral tibial apophysis arc-shaped (vs flattened), the spines on the tip of the cymbium in different positions, the posterior arm of the conductor distinctly hook-like (vs slightly curved) (Figs 10A, B, 11E–G), and the spermathecae pear-shaped (vs irregularly shaped) (Figs 10D, 11I). Additionally, the new species has an undivided cribellum (female) or reduced cribellum (male) (Figs 11C, D), instead of having a reduced cribellum in both sexes, as in *T.relicta*. Besides that, the new species is also similar to *T.lhunzeensis* sp. nov. (Figs 8A–D, 9C–G), but it differs from the latter by the length of the posterior arm of the conductor (as long as the embolus in the new species, vs half the length of embolus in *T.lhunzeensis*), the hook-shaped end of the conductor (vs sharp end) (Figs 10A, B, 11E–G), the large copulatory openings with narrow space between them (vs small, widely spaced copulatory openings), and the S-shaped copulatory ducts (vs comma-shaped) (Figs 10C, D, 11H, I).

##### Description.

**Male (holotype).** Habitus as in Fig. 10A. Total length 6.48 (5.90 in a sole paratype male). Prosoma 3.18 long, 2.35 wide; opisthosoma 3.55 long, 2.35 wide. Eye sizes and interdistances: AME 0.10, ALE 0.13, PME 0.10, PLE 011; AME–AME 0.11, AME–ALE 0.09, PME–PME 0.17, PME–PLE 0.19, ALE–PLE 0.08. MOA 0.34 long, anterior width 0.30, posterior width 0.39. Clypeus height 0.18. Chelicerae with 4 promarginal and 3 retromarginal teeth. Leg measurements: I 8.70 (2.42, 3.21, 1.95, 1.12); II 7.25 (1.99, 2.61, 1.63, 1.02); III 6.17 (1.73, 2.10, 1.43, 0.91); IV 8.61 (2.21, 3.12, 2.22, 1.06). Leg formula: 1423. Dorsum of opisthosoma with blackish cardiac mark. Cribellum reduced.

***Palp*** (Figs 10A, B, 11E–G). Tip of cymbium with 3 spines. Anterior arm of conductor tapering gradually and terminating at about 11:00 o’clock; posterior arm terminating in spiral, distal part as long as embolus, with a hook-shaped tip. Embolus originating at about 9:00 o’clock position.

**Female (paratype).** Habitus as in Fig. 10B. Total length 6.45 (6.33–7.97 in other paratype females). Prosoma 2.69 long, 1.94 wide; opisthosoma 3.88 long, 2.40 wide. Eye sizes and interdistances: AME 0.10, ALE 0.13, PME 0.10, PLE, 0.10; AME–AME 0.11, AME–ALE 0.07, PME–PME 0.15, PME–PLE 0.14, ALE–PLE 0.06. MOA 0.27 long, anterior width 0.29, posterior width 0.35. Clypeus height 0.18. Leg measurements: I 6.45 (1.86, 2.35, 1.40, 0.84); II 5.56 (1.62, 1.94, 1.26, 0.74); III 5.20 (1.55, 1.66, 1.20, 0.79); IV 6.92 (1.88, 2.49, 1.64, 0.91). Leg formula: 4123. Cribellum undivided (Fig. 11C).

***Epigyne*** (Figs 10C, D, 11H–I). Copulatory openings large, almost oval. Weakly sclerotized part of copulatory ducts S-shaped, nearly forming a circle first and then extending posteriorly, about 3 times longer than length of strongly sclerotized part. Spermathecae small, pear-shaped.

##### Distribution.

Known only from the type locality, Xinjiang, China (Fig. 16).

#### ﻿ Tricholathys serrata

Taxon classificationAnimaliaAraneaeDictynidae

sp. nov. (齿状毛隐蛛)

7A706798-0C52-5F2F-8210-8BC090A83215

https://zoobank.org/876FAB03-A845-4859-97D9-8DABBC3FDFD1

Figs 12 , 13 , 16

##### Type materials.

***Holotype* male**: China, Tibet, Gemucuo, 33°39′27″N, 85°49′19″E, September 1990 (SWUC-T-DI-12-01). ***Paratypes***: 2 females (SWUC-T-DI-12-02~03), with same data as holotype.

##### Etymology.

The specific epithet comes from the Latin adjective *serratus*, meaning “serrated” and refers to the shape of the posterior arm of the conductor; the gender is feminine (*serrata*).

##### Diagnosis.

**Figure 12.**
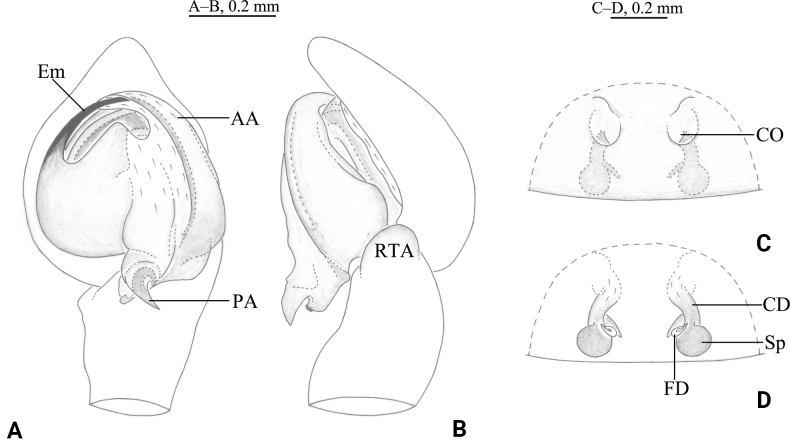
*Tricholathysserrata* sp. nov. **A, B** holotype male **C, D** paratype female **A** left male palp, ventral view **B** same, retrolateral view **C** epigyne, ventral view **D** same, dorsal view. Abbreviations: AA = anterior arm of conductor; CD = copulatory duct; CO = copulatory opening; Em = embolus; FD = fertilization duct; PA = posterior arm of conductor; RTA = retrolaterial tibial apophysis; Sp = spermatheca.

**Figure 13.**
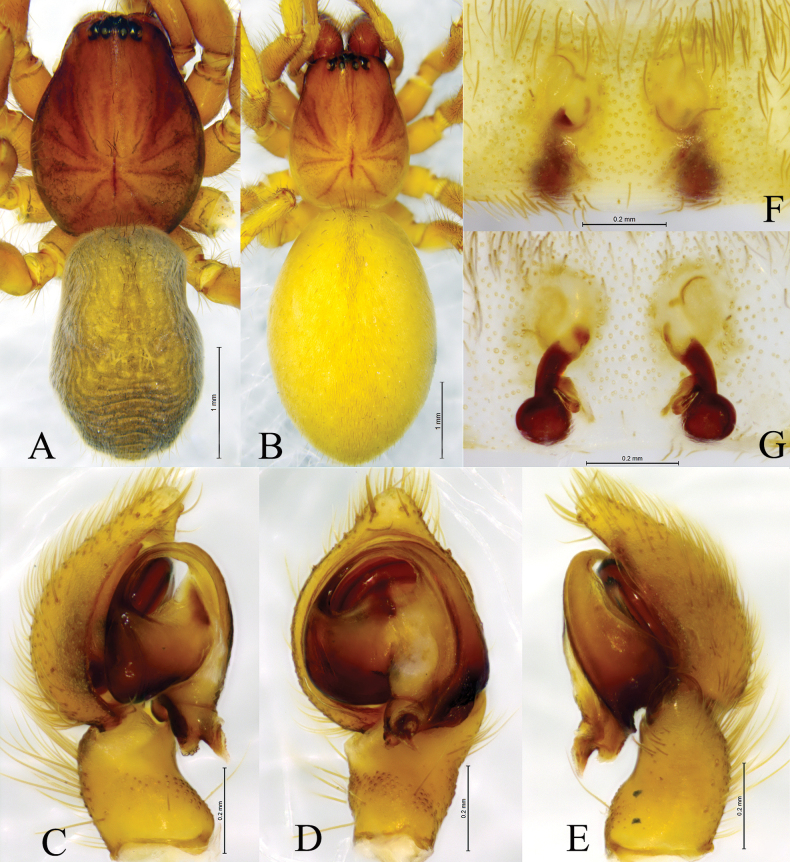
*Tricholathysserrata* sp. nov. **A, C–E** holotype male **B, F, G** paratype female **A** male habitus, dorsal view **B** female habitus, dorsal view **C** left male palp, prolateral view **D** same, ventral view **E** same. retrolateral view **F** epigyne, ventral view **G** same, dorsal view.

This species can be distinguished from all congeners by the bifurcated tip of the posterior arm of the conductor (Figs 12A, B, 13C–E), the very short tibia, the rounded tip of retrolateral tibial apophysis, the short, almost straight copulatory ducts which originate from the mesal part of the epigynal plate, and the spermathecae which is wider than long (Figs 12C, D, 13F, G).

##### Description.

**Male (holotype).** Habitus as in Fig. 13A. Total length 4.04. Prosoma 2.05 long, 1.59 wide; opisthosoma 2.13 long, 1.40 wide. Eye sizes and interdistances: AME 0.09, ALE 0.10, PME 0.07, PLE, 0.07; AME–AME 0.09, AME–ALE 0.04, PME–PME 0.10, PME–PLE 0.09, ALE–PLE 0.05. MOA 0.22 long, anterior width 0.22, posterior width 0.25. Clypeus height 0.10. Chelicerae with 3 promarginal and 2 retromarginal teeth. Leg measurements: I 5.62 (1.60, 2.12, 1.16, 0.74); II 5.06 (1.41, 1.82, 1.12, 0.71); III 4.65 (1.29, 1.53, 1.13, 0.70); IV 5.83 (1.58, 2.01, 1.46, 0.78). Leg formula: 4123.

***Palp*** (Figs 12A, B, 13C–E). Tibia short, as long as wide. Retrolateral tibial apophysis wider than long, with rounded tip. Anterior arm of conductor terminating at about 11:00 o’clock position; tip of posterior arm of conductor bifurcated; ventral branch longer than dorsal; dorsal branch serrated (Figs 12B, 13C, E). Embolus with large base; filamentous part originating at about 9:30 o’clock position.

**Female (paratype).** Habitus as in Fig. 13B. Total length 5.45 (4.86 in other paratype female). Prosoma 2.03 long, 1.59 wide; opisthosoma 3.40 long, 2.24 wide. Eye sizes and interdistances: AME 0.08, ALE 0.10, PME 0.08, PLE, 0.09; AME–AME 0.10, AME–ALE 0.04, PME–PME 0.13, PME–PLE 0.10, ALE–PLE 0.06. MOA 0.26 long, anterior width 0.24, posterior width 0.27. Clypeus height 0.11. Leg measurements: I 5.34 (1.57, 1.90, 1.13, 0.74); II 4.95 (1.41, 1.71, 1.07, 0.76); III 4.72 (1.33, 1.58, 1.03, 0.78); IV 5.69 (1.61, 1.86, 1.40, 0.82). Leg formula: 4123.

***Epigyne*** (Figs 12C, D, 13F, G). Copulatory openings about as large as the spermathecae, separated by a space wider than their diameter. Strongly sclerotized part of copulatory ducts twice as long as length of weakly sclerotized part and with a tapered accessorial caecus. Spermathecae globular, small, diameter slightly more than width of copulatory ducts.

##### Distribution.

Known only from the type locality, Tibet, China (Fig. 16).

#### ﻿ Tricholathys xizangensis

Taxon classificationAnimaliaAraneaeDictynidae

sp. nov. (西藏毛隐蛛)

3809D617-BDC0-5189-A416-21845D25E680

https://zoobank.org/40CC8820-38F6-4CEF-AF07-FD9A6B4E6C9F

Figs 14 , 15 , 16

##### Type materials.

***Holotype* male**: China, Tibet, Coqen County, Meiduo Village, 30°39′19.43′′N, 85°7′54.62′′E, elev. 4751 m, 29 July 2020, L.Y. Wang et al, leg. (SWUC-T-DI-13-01). ***Paratypes*** (1 male and 5 females): 4 females (SWUC-T-DI-13-02~05), with same data as holotype; 1 male and 1 female, Ge’gyai County, 32°31′17.51′′N, 82°28′47.63′′E, elev. 4321 m, 29 July 2020, L.Y. Wang et al. leg. (SWUC-T-DI-13-06~07).

##### Etymology.

The specific name is derived from the type locality the location of the type locality in Tibet; Xizang is a Chinese name for Tibet.

##### Diagnosis.

**Figure 14.**
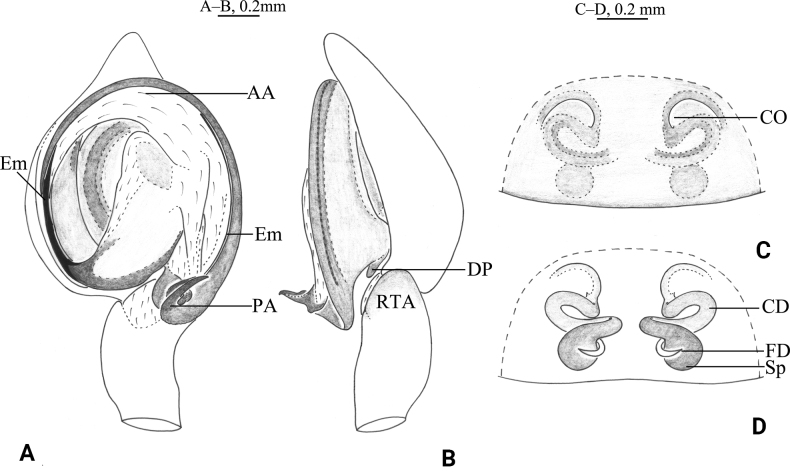
*Tricholathysxizangensis* sp. nov. **A, B** holotype male **C, D** paratype female **A** left male palp, ventral view **B** same, retrolateral view **C** Epigyne, ventral view **D** same, dorsal view. Abbreviations: AA = anterior arm of conductor; CD = copulatory duct; CO = copulatory opening; DP = digitiform process; Em = embolus; FD = fertilization duct; PA posterior arm of conductor; RTA = retrolaterial tibial apophysis; Sp spermatheca.

**Figure 15.**
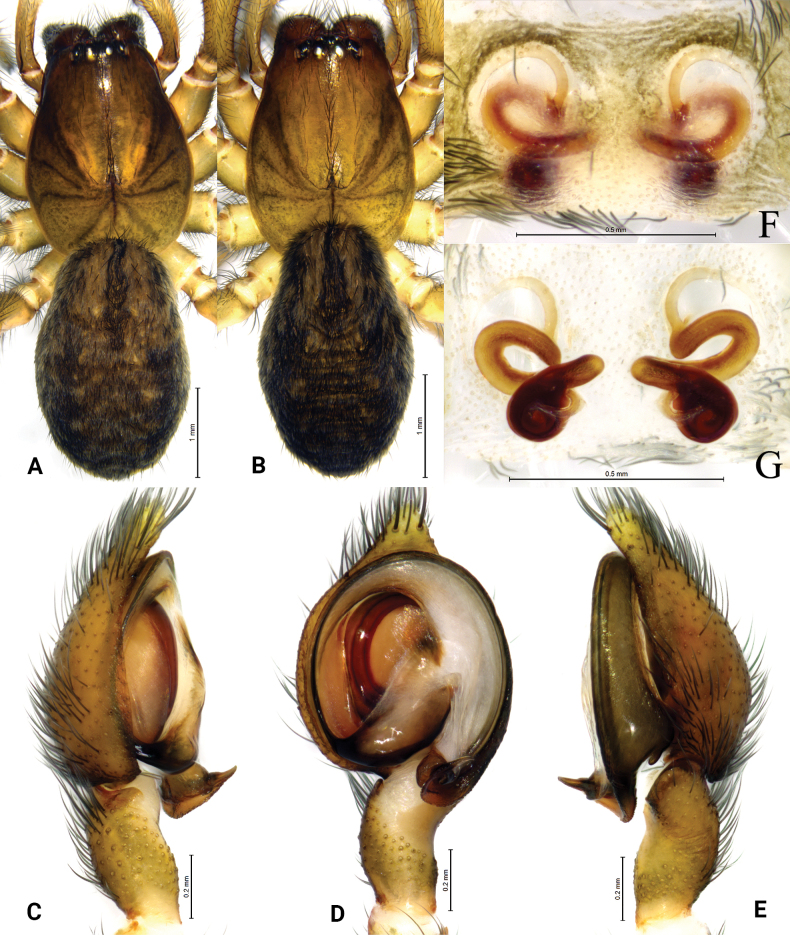
*Tricholathysxizangensis* sp. nov. **A, C–E** holotype male **B, F, G** paratype female **A** male habitus, dorsal view **B** Female habitus, dorsal view **C** left male palp, prolateral view **D** same, ventral view **E** same, retrolateral view **F** epigyne, ventral view **G** same, dorsal view.

This species can be distinguished from all other congeners in having the posterior arm of conductor short and with a hook-shaped end, the anterior arm of the conductor terminating at about the 9 o’clock position, the embolus originated at about 7:30 o’clock (Figs 14A, B, 15C–E), the copulatory ducts long, with 3 turns, and with an indistinct membranous part (Figs 14C, D, 15F, G).

##### Description.

**Male (holotype).** Habitus as (Fig. 15A) total length 4.95. Total length of males 4.95–5.50. Prosoma 2.52 long, 1.90 wide; opisthosoma 2.70 long, 1.74 wide. Eye sizes and interdistances: AME 0.08, ALE 0.11, PME 0.08, PLE 0.11; AME-AME 0.08, AME–ALE 0.06, PME–PME 0.15, PME–PLE 0.12, ALE–PLE 0.04. MOA 0.25 long, anterior width 0.25, posterior width 0.31. Clypeus height 0.13. Chelicerae with 4 promarginal and 2 retromarginal teeth. Leg measurements: I 5.59 (1.63, 2.05, 1.11, 0.80); II 4.93 (1.43, 1.68, 1.07, 0.75); III 4.44 (1.34, 1.41, 0.95, 0.74); IV 5.86 (1.66, 1.96, 1.37, 0.87).

***Palp*** (Figs 14A, B, 15C–E). Tibia with retrolateral apophysis with broad, rounded tip. Tip of Cymbium with 3 spines. Anterior arm terminating at about 9:00 o’clock; posterior arm terminating in digitiform, uncinate tip. Embolus originating at about 7:30 o’clock position.

**Female (paratype).** Habitus as in Fig. 15B. Total length 4.35 (4.35–5.91 in other paratype females). Prosoma 2.26 long, 1.67 wide; opisthosoma 2.48 long, 1.53 wide. Eye sizes and interdistances: AME 0.09, ALE 0.11, PME 0.08, PLE, 0.09; AME–AME 0.09, AME–ALE 0.04, PME–PME 0.14, PME–PLE 0.12, ALE–PLE 0.06. MOA 0.27 long, anterior width 0.27, posterior width 0.31. Clypeus height 0.17. Leg measurements: I 4.88 (1.43, 1.65, 1.08, 0.72); II 4.58 (1.37, 1.52, 1.00, 0.69); III 4.08 (1.16, 1.27, 1.00, 0.65); IV 5.49 (1.49, 1.92, 1.26, 0.82).

***Epigyne*** (Figs 14C, D, 15F, G). Copulatory openings almost semicircular. Spermathecae small. Weakly sclerotized part indistinct; strongly sclerotized part spiral, with 3 turns.

**Figure 16.**
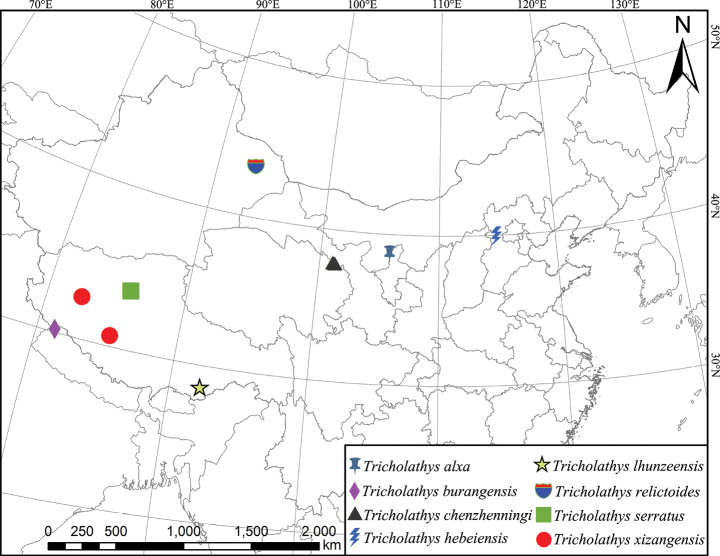
Distribution of *Tricholathys* in China.

**Distribution.** China (Tibet) (Fig. 16).

## Supplementary Material

XML Treatment for Tricholathys

XML Treatment for Tricholathys alxa

XML Treatment for Tricholathys burangensis

XML Treatment for Tricholathys chenzhenningi

XML Treatment for Tricholathys hebeiensis

XML Treatment for Tricholathys lhunzeensis

XML Treatment for Tricholathys relictoides

XML Treatment for Tricholathys serrata

XML Treatment for Tricholathys xizangensis

## ﻿Additional information

### Author ORCIDs

Lu-Yu Wang https://orcid.org/0000-0002-5250-3473

Xian-Jin Peng https://orcid.org/0000-0002-2614-3910

Zhi-Sheng Zhang https://orcid.org/0000-0002-9304-1789

### Data availability

All of the data that support the findings of this study are available in the main text.
